# Translational research needs us to go back to basics and collaborate: interview with Lars Sundstrom

**DOI:** 10.4155/fsoa-2016-0046

**Published:** 2016-07-21

**Authors:** Lars Sundstrom

**Affiliations:** 1 Elizabeth Blackwell Institute for Health Research, University of Bristol & West of England Academic Health Science Network, Bristol, UK

**Keywords:** collaboration, *in vitro*, interdisciplinary science, medicines research, translational research

## Abstract

Lars Sundstrom is Director of Enterprise and Translation at the West of England Academic Health Sciences Network [1] (UK), a Professor of Practice in Translational Medicine and Co-Director of the Elizabeth Blackwell Institute for Health Research at Bristol University [2] (UK), and an honorary Professor of Medicine at Cardiff University (UK). He has extensive experience in translational medicine and clinical neurosciences, holding positions at several eminent universities. He has also held executive and board-level positions at several SMEs, developing new therapeutics for neurological conditions and tools for drug discovery. He has also been an advisor to several UK and local government task forces and to the European Commission and the European Federation of Pharmaceutical Industry Associations. He was a founding member of the European Brain Council in Brussels, and set up the Severnside Alliance for Translational Research, developing a regional network partnership to link clinical and basic scientists. He was also involved in the creation of Health Research Wales.

## Q Can you tell us a little about your career to-date, & what your current roles entail?

My father was an inventor and worked on the equipment that went to look for life on the first Mars expedition with NASA. When I was younger, I spent the summer with him in California (USA) in the laboratory and decided that is what I would like to do when I grew up. So, I went to study biochemistry where I became quite interested in the brain and how it works. I went on to do a PhD and a Postdoc on brain sciences at the University of Oxford (UK). In particular, I became very interested in how drugs affected the brain. I started working on anti-epileptic medication, which led me to work very closely with drug companies and pharmaceutical research.

I then moved to Southampton to become a lecturer in pharmacology, where I continued working on anti-epileptic medication. It was there that I started working closely with clinicians and that is how I got interested in stroke. At that time we were trying to develop better treatments to prevent brain damage in stroke patients. What was different about this research was that the clinicians were telling us that the models we were using in the laboratory – to figure out what molecules might be good at protecting brain cells from damage, – were absolutely useless in the clinic. This was because everything we did in the laboratory was under controlled conditions so it could not ever replicate a real clinical situation. In fact, that whole area of industry failed in the 1980s and 1990s because of this and little research has been done on this since then. Translation of basic science into the clinical situation is very difficult to do!

At that point I became interested in how to generate models and systems that can actually replicate the clinical situation, rather than just the controlled laboratory situation. That led me into what people now call translational research (it was not called that at the time) and I spent a bit of time working in the biotech industry. I left the university for about 5/6 years to go and develop the models we had developed in the academic laboratories to work in an industrial setting. Our aim was to try and scale the models up to be useful for measuring the effects of drugs *in vitro*. Mainly, our experiments involved taking human tissues outside of the body to try and keep them alive long enough to test potential new drugs on them. We found that it worked but it became very difficult to get a reliable source of human tissue, so we then started playing around with stem cells to see if we could make tissues out of them instead.

In 2009 I then moved to the University of Bristol (UK) where I was asked to look after a project that was funded by the Medical Research Council, called the Severnside Alliance for Translational Research (SARTRE). The objective of this project was to try and get people across different universities to cooperate together and work with industry to form a multi-university – industry alliance to develop new therapeutic tools or drugs. I was involved in that for a number of years and it eventually led to me working with a few colleagues of mine to set up the Elizabeth Blackwell Institute (Bristol, UK). We set up teams of people from across different disciplines, for example, engineers and medics, chemists and biologists, to solve problems that need a multidisciplinary approach. We were very lucky that the Wellcome Trust funded a lot of this research. Then, 3 years ago, I was asked to help set up the West of England Academic Health Sciences Network (UK), funded by National Health Service (NHS) England – that is where I am now. We look at the very late stages of translation; at things that are heading for application in the healthcare sector, mainly the NHS. The aim of this project is to speed up the development of new tools to help people. We are also trying to find new ways of getting different groups of people to collaborate in the same way as I mentioned above.

## Q For your work in improving collaboration, do you think you are close to achieving your aims?

I think that some of it is starting to bear its fruit. It is like all research; you do not know what the results are going to be until you do it. You can clearly see a cross-fertilization of one set of knowledge with another – it is very rewarding to see that happen. I have actually just received an email this morning from a group that we funded who were working on intensive care. Their work involved providing clinicians with a way to deal with all of the information they receive about their intensive care patients. They have been working with algorithms that collect all of the patient's vital signs. The algorithms then work out which patient the clinicians should focus on at that precise moment in time, to provide the most effective care. We got them working with psychologists who were experts in how to present the right piece of information at the right time, to a very busy person, to get them to do the right thing – technical as you can imagine! I have just seen the paper they have produced – it is very nice, and has undoubtedly saved a number of lives since last year when it was implemented, so that is quite rewarding [3].

## Q That is really amazing. What would you say are the main obstacles that are still in place to improved collaboration?

I think the biggest problem is that people have different cultures – they speak different languages – and understanding what drives a person is very important. University researchers are very often focused on getting the resources they need to do the research they need to do, and they are usually very clear in their mind about the research that they want to do. When you mention collaboration they sometimes think they will have to step slightly away from what is their immediate concern. In industry, people are usually very focused on productivity. They have the end goal in sight and time is very important to them – it is less so if you are doing basic research. If you are working in the healthcare sector you are mainly interested in quality of care and improving systems. Therefore most of the time, the healthcare sector tries to make very small continuous improvements rather than making radical moves that could potentially be quite disruptive. Culture is very important. Another barrier that we could fix, and which we are not doing very well, is getting our funding systems aligned to allow translation to happen. It is very difficult to move projects across different sectors as there is not a lot of incentive to do collaboration. To fix this would be relatively easy, aligning the funding sources to fund the collaboration rather than the individuals.

## Q Do you think that we will ever overcome these barriers?

I think we should do and there is no reason why we should not. We have experimented a little bit with this on a project that I was involved in setting up called the Bio-E Initiative – in other words, biomedical engineering. We brought together engineers and biomedical scientists across different universities to work as a team. Essentially, we gave each of these groups half the funding, and their collaborator the other half. The researchers needed to come from a different sector and a different university. Automatically, we were then funding multidisciplinary, multi-sector collaborations because they did not receive any money unless they showed true collaboration – we were funding the project rather than the individuals.

## Q You mentioned healthcare & how it can be an issue for those sectors to make small changes to their workflows. You have spoken out about the issues with collaborations between business & the NHS; why do you think that remains such a controversial topic?

I think there are two reasons. The first is that people still think of the NHS as free at the point of need, but there is no such thing as free. The NHS is paid for in a number of ways, one of which is through the tax payers, and most of the tax payers work for businesses. Essentially there is therefore a need to encourage growth in the business sector in order to drive the NHS forward. More closely, there is also a huge opportunity for the NHS to work more productively with businesses. I think that the NHS is really good at buying things from businesses but the concept of collaborating to co-develop things with businesses is quite new to them. It is something that culturally can be very difficult because it means that businesses will profit, and many people that work for the NHS think that that is wrong. In my view it is not wrong at all and it should in fact be encouraged, as profit is only a dividend of somebody's time and efforts to get their product on to the market. I think some people in the NHS need to accept more collaborative and cooperative type models for business rather than just thinking transactionally.

## Q Do you think that is the only real way forward for a sustainable NHS?

I think it is possible to have a system that is funded by the tax payer but what the NHS has to do is to become more efficient. Because of the demographics of healthcare and because it is publicly funded in the UK, it is going to be difficult to keep up with increasing rising costs – in fact it is going to be impossible to do so, but I think most people realize that. Therefore the only way forward is to deliver a more efficient system. One way you can definitely do this is to encourage innovation, bringing in technologies that make it easier. I have always said that mobile health is a great example of that; people could manage their own conditions with mobile technology rather than having to rely on the professionals all the time. We are looking at a sustainable NHS where people are taking much more control of their own health and using their own tools to do that. These are maybe tools they can buy themselves or that are provided to them for a period of time on the NHS. I think it is up to all of us to take that responsibility of managing our own conditions and the conditions of people around us more effectively. We cannot just expect that the system will always provide for us, because it would not be able to do that.

## Q You mentioned mobile health. What is happening in this field at the moment that is exciting you?

At the moment I am part of a rather large initiative called an NHS Test Bed, which kicked off at the beginning of May in the West of England [4]. We are getting people with diabetes to use online tools for education while also providing the tools to allow them to manage their condition more efficiently. We are essentially asking people to sign up to platforms and try out various self-management tools – things that can remind patients to control their diet better, and when to take their medication. A good example is from one of the companies that we are working with, which have an application where you can Skype™ your dietitian if you are unsure about anything. You can use video conferences to discuss anything at that precise moment in time. These are the sorts of technologies that are available right now.

This initiative is quite unique in that we have ten companies; a charity, Diabetes UK; and ten NHS provider organizations and commissioners and we are all working together to try and solve this across a rather large scale. We are hoping that once the platform is fully up and running it will have about 12,000 people signed up. This creates a huge potential saving to the NHS over time as at present it is one of the conditions that are costing the NHS the most amount of money. If we can just make a small dent in that we will make a huge cost saving and people will live healthier lives as well. In addition, technology companies will see their business grow. So it is win, win, win!

## Q That sounds excellent. Returning to your earlier discussion regarding translation, in an ideal world what would you like to see from the *in vitro* research models of the future?

*In vitro* research is obviously the area I have been working on for a good deal of my life. As I mentioned, I initially got into this research because clinicians were telling us our laboratory models were completely useless in practice. Thus, the research models of the future actually need to replicate some of the situations that clinicians find themselves in. A good example of this might be in cancer treatment. Up until now we have been using very refined models that we control. This is very difficult to do because people have different genetic backgrounds so will respond differently to drugs. Therefore what we need to do is to be able to replicate all of the variability of the human into the *in vitro* models. However, that of course provides a bit of a problem, because if you have variability in the system that you are testing, to reflect the variability in humans, then you almost have to do clinical trials *in vitro* to get the same result. Would it not be smarter to do it on a rather large scale, relatively quickly, *in vitro*, rather than actually doing it on a large population of people? What an ideal model should do is to make the drugs we put into people more effective and more selective to the right patient. Once we have done the *in vitro* studies we should be able to work out which drugs work against which cell or gene background. We should then be able to determine that on the individual themselves and say “well, the right treatment for you is actually this one”. That is a form of precision medicine, if you like, and I think the *in vitro* systems have not really caught up with this yet and that is what I would like to see happen.

## Q So how far do you think we are from that?

Not far at all. At the moment we can replicate these things on a fairly small scale. What we find difficult to do is to replicate them on an industrial scale. The future really needs us to develop platforms that allow us to test our models on a sufficient scale before doing it in humans. At the moment, because we can only do it on a small scale it is also fairly expensive, so we need to drive the cost down by making it a larger scale parallel-type platform. Some of the things I have seen recently have been quite optimistic and I feel we are quite close to that now. The cost is still quite high but that is always the case with new technology. Once the technology is more widely used the cost will go down.

## Q On the subject of cost, the billion dollar question: if you had unlimited funding what would you do with it?

Well funnily enough this did actually happen to me once. I was invited to dinner with a very prominent philanthropist. I was not quite sure why I was invited but after we sat down he said “I have invited you all here as you have all come to tell me why I should invest heavily in brain research”. I then realized looking around the table that we all represented different sectors of this research. He explained how he was willing to put one billion pounds on the table if we could each tell him what we thought the most important research area was. About ten people all stood up and said their piece. At the end he said "all of you people have said different things so I am not going to put any money on the table. What you need to do as a community is agree between yourselves on what the most important priorities are and people will give you money." That was a really interesting experience. What would I personally do if I had unlimited funding and why? My heart has always been in brain research; it has always been my main frontier of knowledge. I personally would give the money to basic science rather than translational science because we understand so little about how the brain works. We still have no idea why we sleep, and that is quite fundamental. We only understand a fraction of what we need to in order to combat some of these major illnesses. The thing about brain disorders is that the brain is who we are. If you lose that, you lose cognition and the ability to be who you are and then you are just a shell, you are just a body. So it is vitally important.

## Q Do you think we should move more toward making a priority list for research?

We kind of do already. I think, for example, there are some areas that are going to fit more with public funding than with private sector. There are some good examples of this in research on brain disorders where it has become very difficult for industry to make a return. For example in stroke patients, there is very little research on what happens immediately once you have had a stroke. There are ways to prevent it and ways to treat it after but very little is known on how to stop the effects while they are happening. This is because it is unlikely companies will be able to make a return on this area of work. Therefore, it is given to the public sector. Another great example is antibiotic resistance. Drug companies would find it very difficult to make the sufficient return they need to do this kind of research, so again we are looking at large-scale global initiatives to improve this. I think having large projects with multiple teams to develop research is the way to go. The question is how you build the required infrastructure. It is a bit like saying you want to go to the moon, and getting all the individual researchers to work in isolation and hope that everything is going to come together – it is unlikely. You need a ‘mission control’ to ensure everything fits together, which is very different to how we do science. Translational research should be a coordinated effort.

## Q Finally, do you think the need to go back to the beginning with research is reflected across all the medical sciences?

I think so; without research there is nothing really to pin new discoveries on. We need basic science to understand how to translate things. What is misunderstood is that there is no direct correlation between curiosity-driven research and applied translational research. Of course the two are related – without curiosity-driven research you cannot develop translational research, but there is not a linear relationship between a piece of work and its output. In other words, when you create knowledge someone else will pick up this research and do things you have never even thought of with it. As individual knowledge increases it also increases globally. In my opinion the people who do the basic science are very different to the people that do the translational research and you need different skills to do both. It is wrong to think that you can cut basic science at the expense of translational science – you need to have both and they both need funding.
